# Boron Nitride–Titania Mesoporous Film Heterostructures

**DOI:** 10.1021/acs.langmuir.1c00460

**Published:** 2021-04-21

**Authors:** Junkai Ren, Luigi Stagi, Luca Malfatti, Sebastiano Garroni, Stefano Enzo, Plinio Innocenzi

**Affiliations:** Laboratory of Materials Science and Nanotechnology (LMNT), Department of Chemistry and Pharmacy, CR-INSTM, University of Sassari, Via Vienna 2, 07100 Sassari, Italy

## Abstract

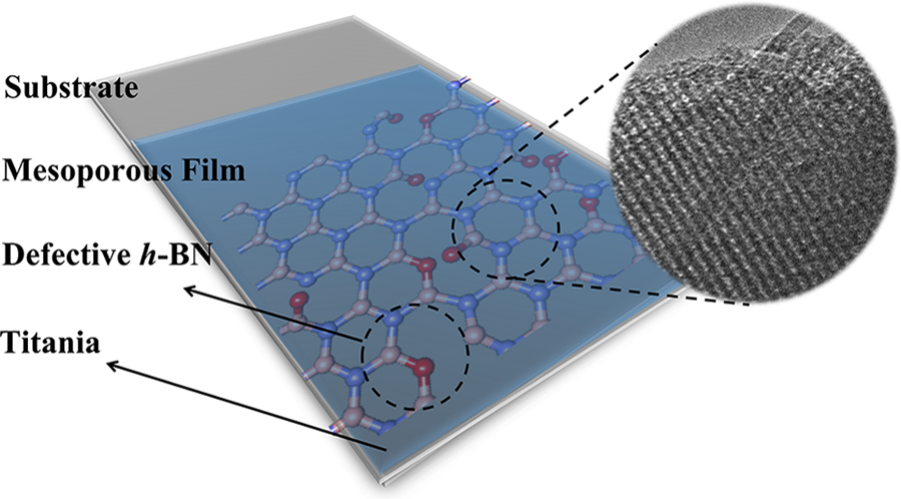

The fabrication of optically active heterostructures in the shape of mesostructured thin films is a highly challenging task. It requires an integrated process to allow in one-step incorporating the two-dimensional materials within the mesoporous ordered host without disrupting the pore organization. Hexagonal boron nitride (BN) nanosheets have been successfully introduced into titania mesoporous films using a template-assisted sol–gel synthesis and evaporation-induced self-assembly. Two types of BN sheets have been used, with and without defects, to investigate the role of defects in heterostructure properties. It has been found that the defects increase the ultraviolet radiation A (UVA) absorbance and enhance the photocatalytic response of the film. The BN sheets are optically transparent and do not exhibit any photocatalytic property but contribute to anatase crystallization via heterogeneous nucleation.

## Introduction

Hexagonal boron nitride (h-BN) is a layered material structurally analogous to graphite with good thermal and chemical stability.^1^ It is considered an ultrawide-band gap semiconductor with a ∼6 eV energy gap and is optically transparent in the visible range.^2^

BN nanosheets (BNNSs) have been produced using different top-down and bottom-up techniques, which allows obtaining two-dimensional (2D) h-BN materials of a few layers.^3^ A crucial issue on the synthesis is controlling the defects. Surface hydroxylation, for instance, induces significant changes in the properties, such as solubility, optical absorption, and luminescence.^4^ Therefore, the assessment of the state of defects is a mandatory step to fabricate heterostructures whose properties depend on the combination of optically active materials for photocatalysis or optoelectronics.^5^

h-BNNSs have been combined with semiconductors, such as TiO_2_, SnO_2_, and InS_2_, to enhance the photocatalytic response.^6,7^ For this purpose, several types of nanocomposites have been built mostly in the form of micro- or nanoparticles.^8^ However, the recent European Union’s classification of titania powders as a category 2 carcinogen by inhalation has severely limited applications based on titania micro- and nanoparticles.^9^ Even research on the lab scale must be performed in highly controlled conditions. An attractive alternative, which does not pose health risks, is preparing BN-based titania heterostructures in the form of thin films. The synthesis of these materials via bottom-up soft-chemistry routes avoids using titania nanoparticles and allows the fabrication of BN–TiO_2_ heterostructures. The possibility of preparing such heterostructures using a porous layer is particularly challenging. Mesoporous titania thin films are a highly efficient platform for photocatalysis and sensing because of the high surface area, the organized porosity, and the control of the titania crystalline state.^10^

In a previous work, we have demonstrated that it is possible to incorporate graphene layers in titania mesoporous films without disrupting the pore self-organization.^11^ This process could be potentially extended to the fabrication of several types of heterostructures formed by a mesoporous matrix and a 2D material. In particular, a template-assisted one-pot process should also allow producing h-BN–TiO_2_ heterostructures formed by BN sheets dispersed in a mesoporous matrix. Fabricating robust heterostructures in the shape of thin porous films allows the design of advanced materials with highly tailored properties, extending the possible fields of utilization.^12^

The combination of titania and h-BN is very effective, and several applications have been envisaged, such as water splitting and photocatalysis.^13^ In particular, much work has been dedicated to producing h-BN/TiO_2_ nanocomposites for photocatalytic applications even if the intrinsic properties of h-BN do not suggest BN as a first choice to combine with titania. The experimental results reported in the scientific literature do not give a coherent picture yet in terms of physical–chemical properties, and it appears that the functional contribution of h-BN to the heterostructure depends on the synthesis conditions and the presence of defects.^14,15^

In particular, the synthesis of heterostructures composed of 2D materials and mesoporous thin films is a challenging task that is still mostly unexplored. The first step is developing a synthesis that allows incorporating the 2D structures into the films without disrupting the pore organization. One-pot routes, where the 2D materials are directly dissolved in the precursor sol, are the most feasible because all of the fabrication can be performed through an integrated process. The main condition to succeed is the solubility of the 2D materials in the solvent employed for the template-assisted sol–gel process. We have successfully prepared for the first time in the present work, to our knowledge, an h-BN–TiO_2_ heterostructure using a mesoporous titania thin film as a matrix. The role of h-BN nanosheets in the synthesis has been investigated, while the response of the heterostructure to ultraviolet (UV) light irradiation has been studied using h-BN sheets with and without defects.

## Experimental Section

### Chemicals

The chemicals used in this study are as follows: hexagonal boron nitride powder (h-BN, 99.5%, Alfa Aesar), deionized water, *N*-methyl-2-pyrrolidone (NMP, 99.5%, Sigma-Aldrich), titanium(IV) chloride (TiCl_4_, Aldrich, 99.90%), ethanol (EtOH, Sigma-Aldrich, 99,5%), Pluronic F-127 (∼12 600 g mol^–1^, Aldrich), and stearic acid (Sigma-Aldrich, 97%).

### Preparation of h-BNNSs

Both defective and defect-free BN sheets were obtained by a sonication-assisted liquid-phase exfoliation method.

Defective h-BNNSs (h-BNNS(d)) were prepared by a water-assisted exfoliation method, according to our previous report.^16^ Briefly, exfoliation was carried out by dispersing h-BN (20 mg) into water (20 mL). After 15 h of sonication, h-BNNSs were collected from the supernatant by centrifuging at 8000 rpm for 10 min.

Defect-free h-BNNSs were obtained by dispersing h-BN (20 mg) into NMP (20 mL). After 15 h of sonication, h-BNNSs were collected from the supernatant by centrifuging at 5000 rpm for 10 min.

The solid samples were obtained by vacuum filtration and then dispersed in EtOH (5.0 mg mL^–1^) for the next step. Their characterization is shown in Figure S1.

### Synthesis of Titania Mesoporous Thin Films and BN–TiO_2_ Heterostructures

The mesoporous titania films were obtained by a template-assisted synthesis using our reported method.^11^ First, the precursor sol was prepared by adding TiCl_4_ (2.2 mL) into EtOH (46.8 mL) with a block copolymer, Pluronic F-127 (1.3 g). After 15 min of magnetic stirring, distilled water (3.6 mL) was added to the mixture. The final molar ratio was TiCl_4_/EtOH/F-127/H_2_O = 1:40:0.005:10. Then, BNNS solutions (600 μL) were added into the titania precursor sol (6 mL).

The concentration of BNNS (1 mg mL^–1^) in the precursor sol is the maximum allowed to obtain optically transparent mesoporous titania films. At higher concentrations, a precipitate forms and the films become opaque.

Silicon wafers and silica glass slides were used as substrates for dip-coating at a withdrawal rate of 10 cm min^–1^. The substrates were immersed in the h-BNNS sol and kept for 30 s before extraction. The relative humidity (RH) was kept under 30% by a dried airflow.

The mesoporous films (TiO_2_, TiO_2_–BNNS, and TiO_2_–BNNS defects) were first dried at 60 °C in air for 10 h and then were thermally annealed in air for 1 h at different temperatures, 300, 350, and 400 °C.

### Characterizations

Transmission electron microscopic (TEM) images were obtained by a FEI Tecnai 200 microscope working with a field emission electron gun operating at 200 kV.

Fourier-transform infrared (FTIR) analysis was carried out by an infrared Vertex 70 interferometer (Bruker). The absorption spectra were recorded in the 4000–400 cm^–1^ range with a 4 cm^–1^ resolution. The baseline was fitted by a concave rubber band correction with OPUS 7.0 software.

Raman analysis was performed by a Senterra confocal Raman microscope (Bruker) with a 785 nm laser excitation, 1 mW power, and 100× objective. The spectra were collected in the 65–1555 cm^–1^ range with a 3–5 cm^–1^ resolution.

Ultraviolet–visible (UV–vis) absorption spectra were collected by a Nicolet Evolution 300 UV–vis spectrophotometer (Thermo Fisher) with a bandwidth of 1.5 nm.

X-ray diffraction (XRD) patterns of thin films were collected in grazing-incidence geometry using a Bruker D8 Discover diffractometer under irradiation with a Cu Kα_1_ line (= 1.54056 Å); the X-ray generator worked at a power of 40 kV and 40 mA. The patterns were recorded in 2θ ranging from 20 to 60° with a step size of 0.02 Å.

The contact angle of the films was measured by an OCA 20 system (DataPhysics) with 4 μL of water droplets deposited on the surface of the films.

Spectroscopic ellipsometry (α-Wollam) with fixed angle geometry was used to measure the thickness and refractive index of the films. The medium square error (MSE) was kept below 30. The residual porosity of the films was calculated according to the ellipsometry analysis data. In detail, the Cauchy parameters were first calculated using dense titania films deposited on silicon, which were prepared via the same route as mesoporous films without the presence of Pluronic F-127. Then, residual porosity values of mesoporous films were calculated using an effective medium approximation (EMA) method via CompleteEASE 4.2 software.

A UV lamp (Spectroline) was used for evaluating the photocatalytic activity. The model is ENF-280C/FE, operating at 230 V, 50 Hz, and 0.17 Amps.

### Evaluation of Photocatalytic Activity

Stearic acid (see the vibrational spectrum in Figure S2) has been used as the molecular probe to evaluate the titania mesoporous films’ photocatalytic activity. The change of the 2945–2840 cm^–1^ vibrational modes, corresponding to −CH_3_ and −CH_2_ stretching, respectively, has been used for evaluation.^10^ The process has been quantified by the corresponding integral of the infrared bands as a function of the irradiation time. First, stearic acid was dissolved in ethanol (concentration, 3.3 mg mL^–1^). Then, the solution (100 μL) was deposited on the films by spin-coating at 1500 rpm for 30 s. The samples covered by stearic acid were irradiated under 365 nm light from the UV lamp at a distance of 100 mm. The radiation time was fixed from 1 to 120 min, and FTIR spectra of these samples were recorded immediately after illumination.

## Results and Discussion

Understanding the interaction between low-dimensional materials and metal oxide semiconductors in heterostructures is fundamental for exploiting such nanocomposites. 2D h-BN represents a particular example of a nano insulator, with a weak p-type characteristic at high temperature (>700 K) and high resistivity (∼1.6 × 10^3^ Ω·cm at 790 K).^17^ Besides, bulk h-BN has a wide optical band gap (∼6 eV).^2^ The energy gap is affected by the preparation method, particularly by the bulk exfoliation used to produce few h-BN layers.^3^ Correspondingly, defect-free h-BN nanosheets are expected to behave as inert systems without significant contributions to physical and chemical phenomena, which involve light absorption in the visible range and charge separation during photocatalysis. Introducing vacancies or doping atoms (e.g., oxygens or carbons) is one of the most suitable strategies to promote UV and visible light absorption and possibly interaction with metal oxides in a heterostructure. In light of this, managing the formation of defects is fundamental to control the nanocomposite properties. The defect control would allow realizing effective h-BN–TiO_2_ heterojunction heterostructures.

In the present work, we have used photocatalysis to unveil the mechanisms governing h-BN–TiO_2_ interactions fostered by defective h-BN nanosheets. For this purpose, we have produced heterostructures in the form of thin films integrating exfoliated h-BN into mesoporous TiO_2_ films. Two different h-BNs have been considered for this work, namely, exfoliated h-BN (BNNS) and oxygen-defective exfoliated h-BN (BNNS(d)) nanosheets (see the Experimental Section for more details).

**Figure 1:**
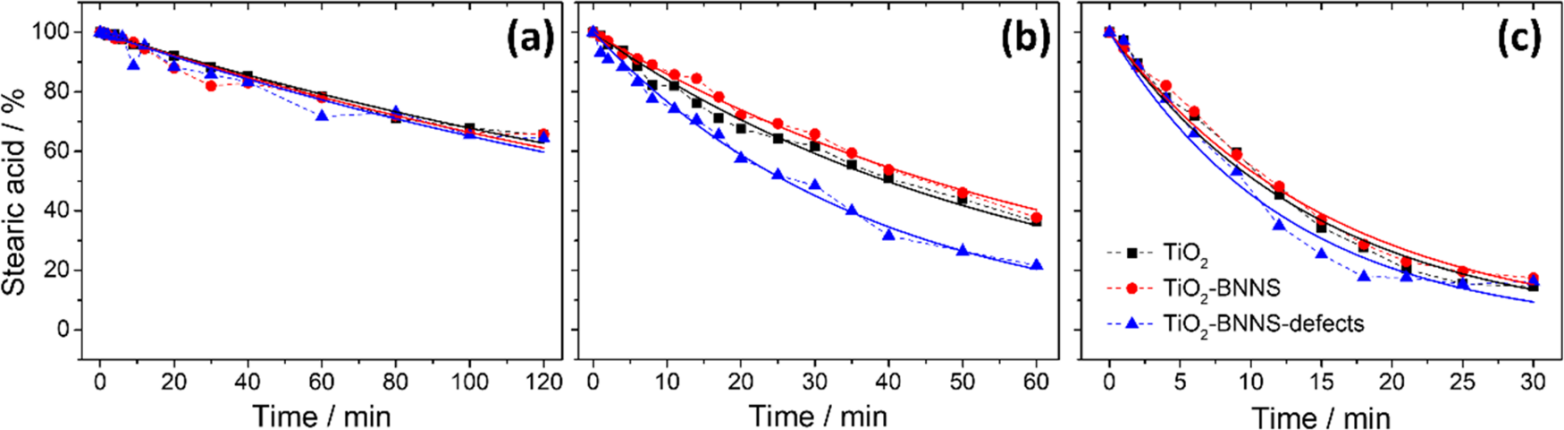
Photodegradation of stearic acid onto TiO_2_ (black line), TiO_2_–BNNS (red line), and TiO_2_–BNNS(d) (blue line) films treated at (a) 300, (b) 350, and (c) 400 °C, respectively. The dots are the collected data via FTIR, and the solid lines are the fitting results.

Stearic acid has been used to evaluate the photocatalytic activity by monitoring the FTIR absorption intensity of the −CH_3_ and −CH_2_ stretching modes. Figure 1 shows the photoinduced degradation of stearic acid produced by different mesoporous films. In the graphs, we have reported the intensity of infrared absorption *I*_t_ as a function of UV irradiation time *t*; *I*_0_ corresponds to the original value before exposure, at *t* = 0 min. The photoinduced degradation of stearic acid (stearic acid/%) has been used to evaluate the photocatalytic activity using the following formula: stearic acid/% = *I*_t_/*I*_0_ × 100%. Reference curves for the photocatalytic degradation of stearic acid as a function of UV illumination time have been obtained using silicon and bare or defective BNNSs as surfaces.

The intensity of stearic acid infrared band does not decrease after 35 min of UV light exposure on silicon; the same trend has been observed if stearic acid is deposited onto the BNNS and BNNS(d) films (Figure S3). This suggests that the h-BN sheets do not show any significant catalytic activity if they are not integrated to form a heterostructure as would be shown hereafter. The photodegradation data can be fitted by an exponential decay law: *I*(*t*) = *I*_0_e^–*kt*^, where the parameter *k* represents the degradation rate. The solid curves in Figure 1 are the fittings, while the calculated *k* values are listed in Table 1.

**Table 1:** Degradation Rate (*k* Values, min^–1^) of the Mesoporous Titania Samples after Annealing at Different Temperatures

mesoporous titania films	300 °C	350 °C	400 °C
TiO_2_	0.00389	0.0175	0.0649
TiO_2_–BNNS	0.00410	0.0151	0.0637
TiO_2_–BNNS(d)	0.00429	0.0266	0.0787

The samples treated at 300 °C show a weak photocatalytic activity with comparable absorbance decays. The degradation follows an almost linear trend, and after 120 min of UV exposure, around 30% of the reference molecule has been degraded. The degradation rate in this case is about 0.004 min^–1^ (±5%) (Figure 1a).

On the contrary, at 350 °C, the three diverse samples show a significant photocatalytic activity and a remarkable difference among them. The *k* value of TiO_2_–BNNS(d) films (0.0266 min^–1^) is 50% higher than those of TiO_2_ and TiO_2_–BNNS films (0.0175 and 0.0151 min^–1^). This demonstrates that BNNS(d) defective structures play an active role in the photocatalytic performance of mesoporous titania. In particular, after 1 h of exposition to UV light, 80% of the stearic acid has been degraded in comparison to 64% of the other samples (Figure 1b).

After annealing in air at 400 °C, the degradation rate increases, but the difference in the photocatalytic response of the 350 °C samples is lower (Figure 1c), as pointed out by the *k* values. The response under visible light has also been tested. In general, the introduction of defects in BN materials may increase the absorbance in the visible range, contributing to the optical response of a heterostructure.^16^ The effect is, however, based on the type and amount of defects; for this reason, holes or large defects are generally introduced in the BN sheets. In our experiments, all of the samples upon illumination by visible light at 450 nm do not exhibit any photocatalytic effect (Figure S4).

Summarizing, the photocatalytic experiments show two main effects: (i) the degradation rate increases with the presence of BNNS(d) in the h-BN–TiO_2_ heterostructure, while the TiO_2_ and TiO_2_–BNNS display the same performance; and (ii) the temperature treatment enhances the photocatalytic activity by improving the transformation of amorphous titania into anatase (vide infra). Furthermore, at higher thermal annealing temperatures, the *k* values tend to coincide regardless of the h-BN structure.

**Figure 2:**
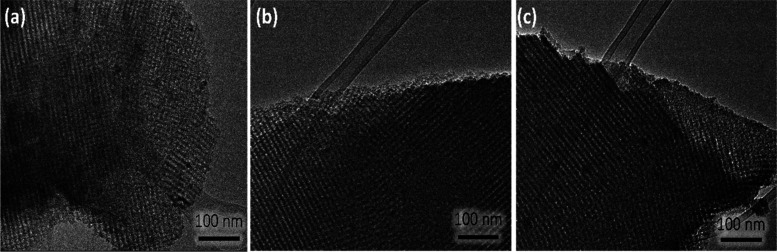
TEM images of the nanocomposite films treated at 350 °C: (a) TiO_2_, (b) TiO_2_–BNNS, and (c) TiO_2_–BNNS defects.

To figure out the impact of BNNS(d) in the heterostructure annealed at 350 °C, we have extensively investigated the structural and optical properties of thin films. Figure 2 shows the TEM images of different mesoporous titania films after thermal treatment at 350 °C. The films exhibit a well-organized mesoporous structure, which is compatible with a body-centered cubic structure with an *Im*3*m* symmetry.^18^ From the surface plot analysis, the wall-to-wall average distances of different types of samples result in 11.74, 11.99, and 11.56 nm for TiO_2_, TiO_2_–BNNS, and TiO_2_–BNNS(d), respectively. Moreover, it is important to emphasize that the addition of h-BN nanosheets, irrespective of bare and defective, does not affect the order and dimension of mesopores.

**Figure 3:**
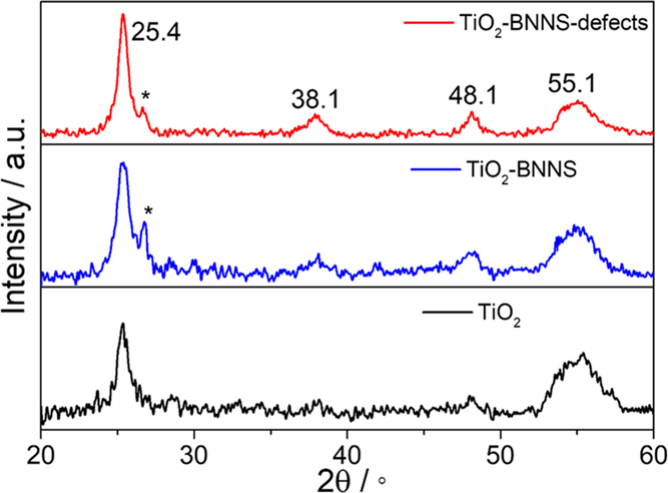
XRD patterns in the 20–60° range in 2θ of the mesoporous titania films upon annealing at 350 °C (undoped TiO_2_, bottom, black line; TiO_2_–BNNS, middle, blue line; and TiO_2_–BNNS(d), top, red line). The symbol * indicates the h-BN phase.

The XRD patterns (Figure 3) of the films are characterized by four diffraction peaks at 25.4, 38.1, 48.1, and 55.1°, corresponding to the (110), (004), (200), and (211) planes of titania anatase (# JCPDS No. 84-1286).^19^ The diffraction peak detected at 26.7° has been assigned to the characteristic (002) reflection in the h-BN phase (# JCPDS No. 01-073-2095).^20^ The signal is clearly detected in the h-BNNS mesoporous titania heterostructures indicating a successful incorporation of the 2D materials.

The titania crystallite size has been estimated by the Scherrer equation^21^*L* = *K*λ/β cos θ, where *L* is the crystallite size, *K* is the Scherrer constant (0.89 is selected here for the integral breadth of the spherical crystal), λ is the X-ray source wavelength (0.154 nm), β is the full width at half-maximum (FWHM, in radians) of the diffraction peak, and θ is the diffraction angle. The main plane (110) at 25.4° has been selected for the calculation of the crystallite sizes; a Lorentz model has been used to fit the FWHM. The corresponding crystallite sizes are 8.4 (TiO_2_), 9.0 (TiO_2_–BNNS), and 10.5 nm (TiO_2_–BNNS defects). The XRD data indicates that the incorporation of BNNSs into the titania mesoporous films promotes the crystallization of titania, as shown by the increase in the crystallite size and the intensity of the diffraction peak. In particular, this effect is emphasized by the presence of defects in the h-BN nanosheets.

**Figure 4:**
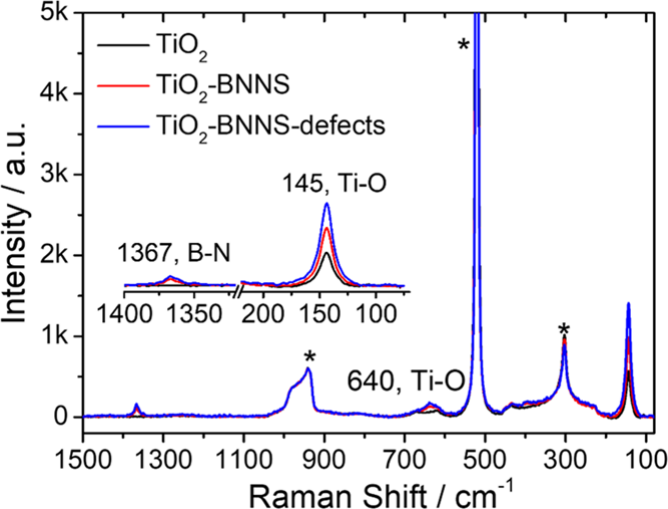
Raman spectra of the TiO_2_, TiO_2_–BNNS, and TiO_2_–BNNS(d) mesoporous films after thermal treatment at 350 °C. Insets show the enlarged spectra in the 1400–1320 and 220–70 cm^–1^ ranges. Raman signals at 300, 520, and 940 cm^–1^ are due to the silicon wafer substrate (*).

Raman spectra of the mesoporous films deposited on silicon wafer substrates support the XRD results (Figures 4 and S5). The weak Raman band at 1367 cm^–1^ is assigned to the G band of h-BN.^16^ This signal appears in all of the thin films regardless of the annealing temperature, indicating the successful incorporation of BNNSs into the titania layers in accordance with XRD. The Raman bands at 145 and 640 cm^–1^ are attributed to the E_g_ vibration mode of O–Ti–O in the anatase phase;^22^ the bands increase in intensity with the temperature treatment (Figure S5b) because of the thermal-induced amorphous-to-anatase transition.^23,24^ At the lowest annealing temperature (e.g., ≤300 °C), the titania films are mainly in an amorphous state and the Raman signal of crystalline TiO_2_ (Figure S5a) remains too weak to be detected.

In accordance with XRD patterns, the presence of BNNSs (both defective and defect-free types) produces an increase in intensity of the TiO_2_ Raman mode, which indicates a higher crystallinity level.^25^

**Figure 5:**
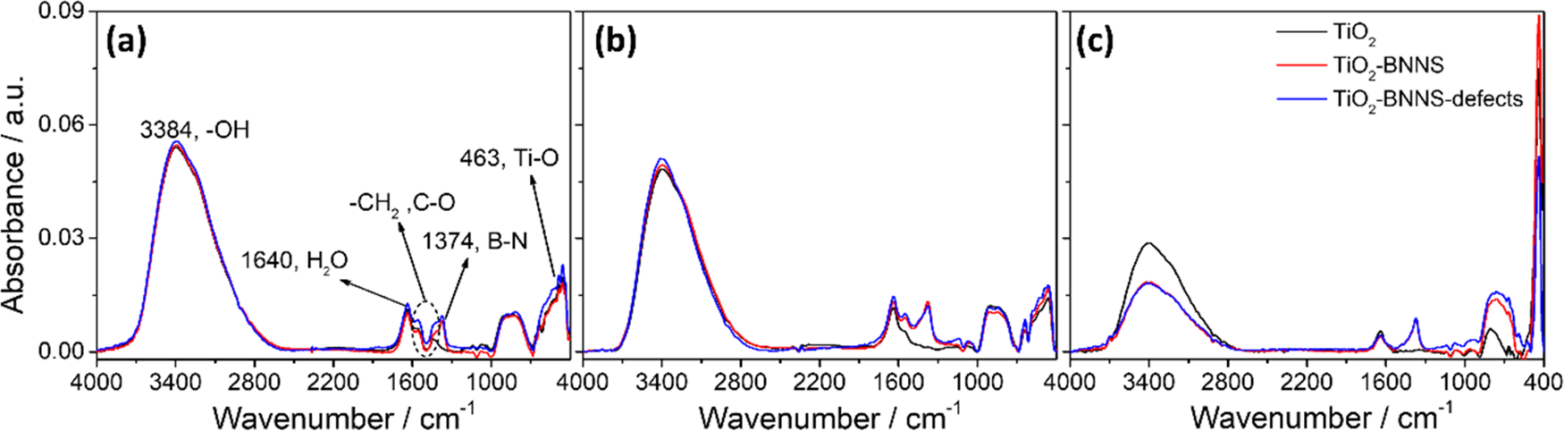
FTIR spectra of the TiO_2_ (black line), TiO_2_–BNNS (red line), and TiO_2_–BNNS(d) (blue line) films after annealing at temperatures (a) 300, (b) 350, and (c) 400 °C.

The mesoporous films have also been analyzed by FTIR spectroscopy. Figure 5 shows the FTIR absorption spectra of mesoporous titania films (TiO_2_ (black line), TiO_2_–BNNS (red line)), and TiO_2_–BNNS(d) (blue line) after thermal treatment at 300, 350, and 400 °C.

Although Raman spectroscopy and XRD diffraction have proved to be more sensitive to detect the anatase phase, the FTIR data well describe the annealing effect on the hydroxyl groups and surfactant chemical moieties. The removal of the residual template can be monitored by the absorption bands in the 1600–1400 cm^–1^ range, e.g., −CH_2_ and C–O; the spectra show that Pluronic F-127 is completely removed after the thermal treatment at 400 °C.

The spectra at higher wavenumbers (4000–2700 cm^–1^) are characterized by a broad and intense signal due to the overlapping of the two bands assigned to Ti–OH (3384 cm^–1^) and H–O stretching overtone in water (3200 cm^–1^).^26^ These bands decrease in intensity at higher temperatures, indicating the completion of the condensation reaction that forms the Ti–O–Ti network. The deconvolution of the Ti–OH and OH stretching bands has shown that at 400 °C a 68% condensation has been attained in the nanocomposites, while in mesoporous titania it is 49% (Figure S6 and Table S1). At 400 °C, therefore, the condensation of titania occurs to a smaller extent than that in mesoporous films containing the BN sheets, which appear to be promoting the condensation reaction.

The absorption band at ∼460 cm^–1^ is attributed to the E_u_ TO mode of anatase titania;^27^ in the samples treated at 300 and 350 °C, this band is hardly detected, while after thermal treatment at 400 °C, it becomes sharper and more intense. The absorption band at 1374 cm^–1^ that does not change in intensity for all of the samples is assigned to the characteristic in-plane B–N stretching vibration.^16^

The structural characterizations highlight the effect of BN in the titania crystallization process. The results of the three techniques, XRD, FTIR, and Raman, converge in identifying h-BN as the agent that promotes heterogeneous crystallization of titania. This phenomenon is particularly evident for the samples treated at 350 °C where only a partial crystallization of TiO_2_ has been achieved. However, this still does not allow getting a full understanding of the role of defective BNNS in the photocatalytic response. As we have seen, the nondefective BNNS has properties comparable to undoped TiO_2_ at 350 °C, despite the higher crystallinity of the corresponding heterostructure.

**Figure 6:**
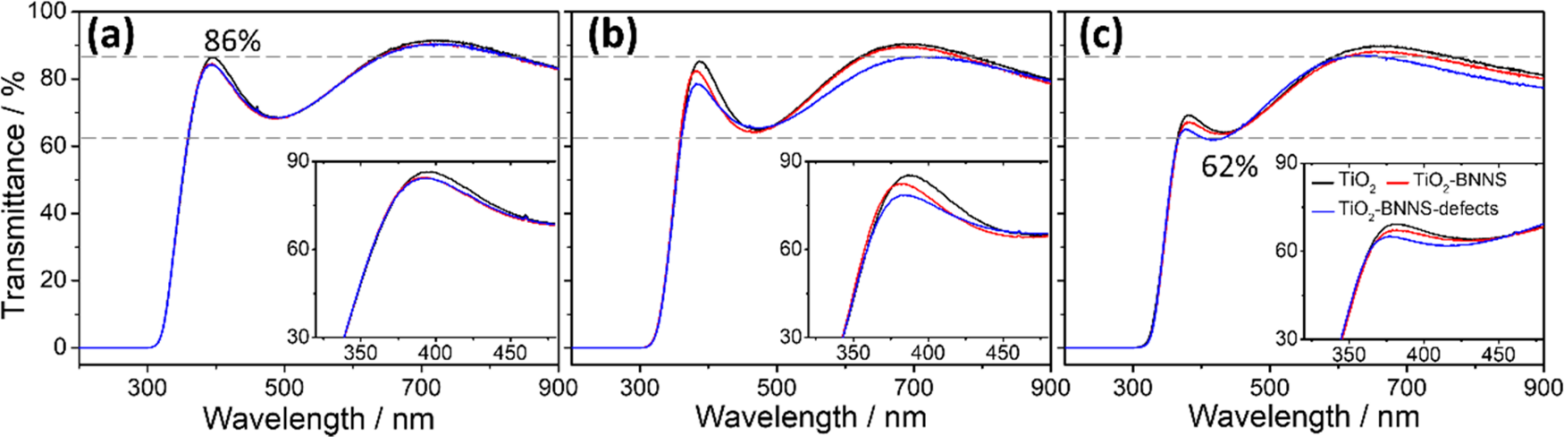
UV–vis transmission spectra of mesoporous films: TiO_2_ (black line), TiO_2_–BNNS (red line), and TiO_2_–BNNS(d) (blue line) after thermal treatment at (a) 300, (b) 350, and (c) 400 °C. The insets show the enlarged spectra in the 320–480 nm range.

Figure 6 shows the UV–vis transmission spectra of the mesoporous films on silica glass substrates. As expected, after annealing at 300 °C, the three different samples show a comparable optical transmittance as expected by the similar photocatalytic performance (Figure 1).

The transmittance at 400 nm decreases with the increase of the annealing temperature with a drop from 86 (300 °C) to 65% (400 °C) for undoped titania. Introducing the BNNSs further results in a transmittance decrease. At the annealing temperature of 350 °C, defective BNNSs in TiO_2_ matrix possess a stronger absorption (e.g., 78.5 vs 82.5%/83.5% at 380 nm in transmittance), which can support the higher photocatalytic effect compared to pristine BNNS. The enhanced absorption arises from the characteristic oxygen defects in the h-BN structure generated by exfoliation in water, as already demonstrated by the theoretical calculation and experimental results in our previous work.^16^ In the visible wavelength range, 500–900 nm, the transmittance in all of the samples remains above 80%, where both the annealing temperature and the presence of BNNSs show a negligible effect on the transmission spectra.

The increase of optical absorption with the temperature and the presence of BNNS(d) is also demonstrated by the band gap (E_g_) values that have been calculated via the Tauc method,^28^ according to the absorption data (see Figure S7). The addition of BNNSs does not change the E_g_ with respect to the undoped samples, while increasing the annealing temperature from 300 to 400 °C shifts the band gap from 3.40 to 3.32 eV.

Spectroscopic ellipsometry has been used to measure the change in thickness and refractive index of the three mesoporous samples treated at different temperatures. Considering an experimental error of around 10 nm, the films have a similar thickness in the range of 155–175 nm (see Table 2), suggesting that the films do not undergo a distinct shrinkage or expansion in a narrow temperature range (300–400 °C) even if they contain the BNNSs. The residual porosity of the films calculated by ellipsometry is 12.1 ± 0.04% (300 °C), 19.6 ± 0.05% (350 °C), and 12.4 ± 0.13% (400 °C).

**Table 2:** Thickness (nm) of TiO_2_ Nanocomposite Films Measured by Spectroscopic Ellipsometry

mesoporous films	300 °C	350 °C	400 °C
TiO_2_	161.7 ± 0.28	158.2 ± 0.46	169.8 ± 0.34
TiO_2_–BNNS	164.5 ± 0.21	179.1 ± 0.30	172.1 ± 0.50
TiO_2_–BNNS(d)	168.5 ± 0.23	165.8 ± 0.17	154.6 ± 0.70

**Figure 7:**
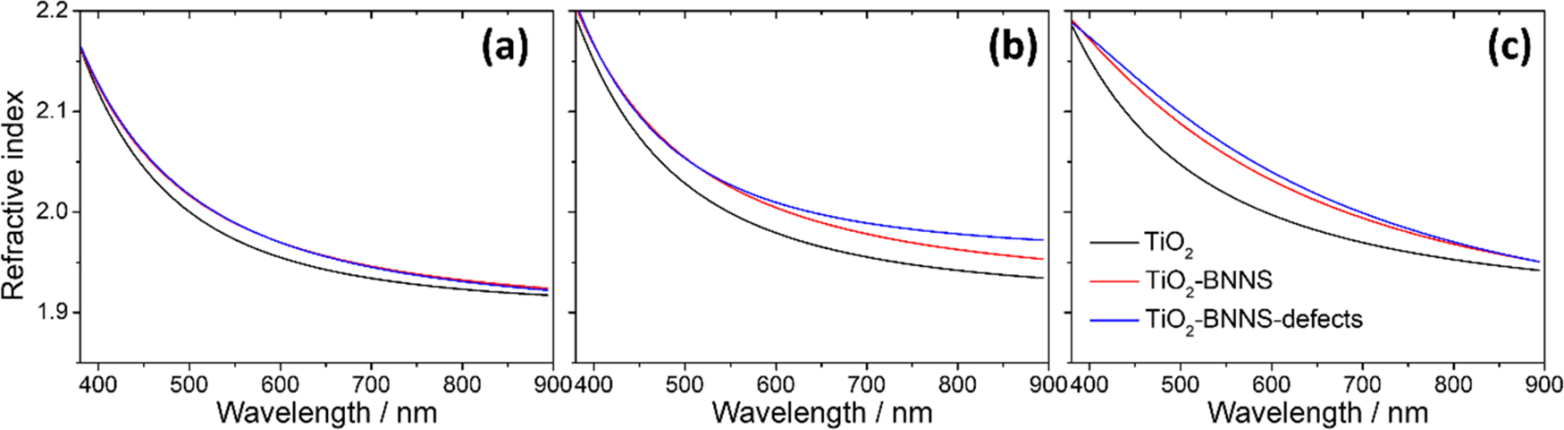
Refractive index (*n*) as a function of wavelength of the TiO_2_, TiO_2_–BNNS, and TiO_2_–BNNS(d) films after thermal treatment at (a) 300, (b) 350, and (c) 400 °C.

Figure 7 shows the dispersion of the refractive index (*n*) in the 380–900 nm range of the films; the *n* values at 633 nm are listed in Table S2. The refractive index increases with the annealing temperature and is higher in the heterostructured mesoporous titania films. On the one hand, the increase of the refractive index can be partly attributed to the increase of pore dimension^29^ because the thermal treatment at higher temperature can remove all surfactant templates (see FTIR spectra in Figure 5). On the other hand, the increase of the refractive index can result from the increased degree of crystallinity,^30^ which has been proved by XRD and Raman results.

We have performed contact angle measurements to investigate the surface wettability of the three different films as a function of temperature treatments (Figure S8). With the increase of the annealing temperature, the contact angle decreases, indicating that the film surface is becoming progressively more hydrophilic. For example, the contact angle of the TiO_2_ film is 43.4° after firing at 300 °C, which decreases to 39.1° at 350 °C and 15.2° at 400 °C. Remarkably, the 400 °C undoped mesoporous film has a smaller contact angle with respect to the samples doped with BNNSs; this difference is derived from the hydrophobic nature of BN^31^ that prevails with the crystallization of anatase. In fact, the contact angle measured on a h-BN material is typically around 50°,^32,33^ which is higher than the value of titania mesoporous films. The decrease of contact angle with annealing and the larger hydrophilicity is, therefore, correlated to the titania crystallization.^34^ However, it is worth noting the lower hydrophilicity value of TiO_2_–BNNS(d) at 300 and 350 °C. This indicates the substantial concentration of hydroxyl defects brought by the defective BNNS, which explains the higher hydrophilicity of the heterostructure.

The experimental results show that exfoliated h-BN does not exhibit any photocatalytic effect under UV (∼365 nm) irradiation. Introducing a high percentage of structural defects in BNNSs, such as vacancies and foreign atoms, is a strategy to make the BN sheets photoresponsive. Using defective BNNSs increases the absorbance in the ultraviolet radiation range (UVA) and the hole promoted reactions in photocatalysis. The impact of defective structures in BNNS depends on the annealing temperature of the heterostructure. At 400° C, the transformation from amorphous titania to anatase governs all of the light-triggered catalysis mechanism, and the state of BNNSs does not affect the optical properties in a significant way. This is not the only effect induced by BNNSs in TiO_2_–BN heterostructures. The presence of h-BN nanosheets promotes, in fact, the nucleation of the anatase phase in the films by lowering the energy barrier to heterogeneous nucleation.

The present results clearly show that h-BN 2D materials do not have any photocatalytic property even if they have been widely used in other works to produce optically active heterostructures with titania.^8,13,15^ Only defective h-BN layers may be used for such a purpose, but controlling such types of defects to assure the level of reproducibility necessary for technological exploitation of the device is not at present realistic. 2D h-BN materials do not appear, therefore, a good choice to form heterostructures for photocatalysis in combination with titania.

## Conclusions

BNNS–TiO_2_ mesoporous nanocomposite films have been fabricated via a template-assisted self-assembly process, and h-BN nanosheets have been successfully incorporated within the mesoporous films without disrupting the pore order. The incorporation of BNNS into a mesoporous film promotes the heterogeneous crystallization of titania into anatase. The incorporation of defective BNNS into titania mesoporous films produces an increased photocatalytic response, which is due to a higher UV absorption. On the other hand, illumination upon visible light does not produce any photocatalytic effect.

The fabrication of optically transparent BNNS–TiO_2_ films via deposition from a liquid phase has been a viable route for controlling the heterostructure’s chemical–physical properties.

## Supporting Information Available

FTIR spectra of h-BNNSs and stearic acid, Raman spectra of the films after treating at 300 and 400 °C, UV–vis absorption spectra, contact angle images (PDF)
