# Supplementary material for: Boron Nitride–Titania Mesoporous Film Heterostructures
Source: Langmuir. 2021 Apr 21;37(17):5348–55. doi: 10.1021/acs.langmuir.1c00460 (PMC8280735; doi:10.1021/acs.langmuir.1c00460)
Supplement: Supplementary file 1 — la1c00460_si_001.pdf [file la1c00460_si_001.pdf]

Supplementary Information

Boron Nitride-Titania Mesoporous Film Heterostructures

Junkai Ren, Luigi Stagi, Luca Malfatti, Sebastiano Garroni, Stefano Enzo and Plinio Innocenzi**

Laboratory of Materials Science and Nanotechnology (LMNT), Department of Chemistry and Pharmacy, CR-INSTM, University of Sassari, Via Vienna 2, 07100, Sassari, Italy.

**Corresponding Authors E-mail: lstagi@uniss.it; plinio@uniss.it*

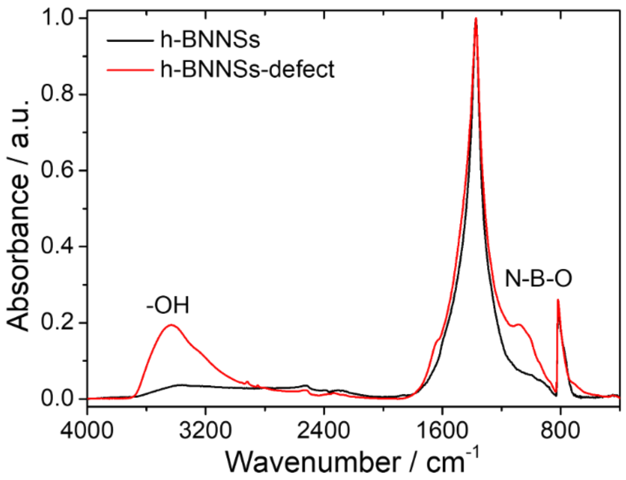

Figure S1. FTIR spectra of NMP-derived h-BNNSs (without defects) and water-derived h-BNNSs (with defects).

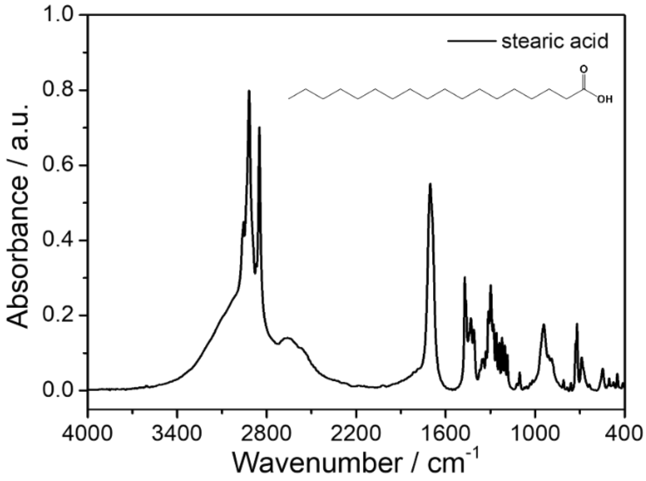

Figure S2. FTIR spectra of stearic acid.

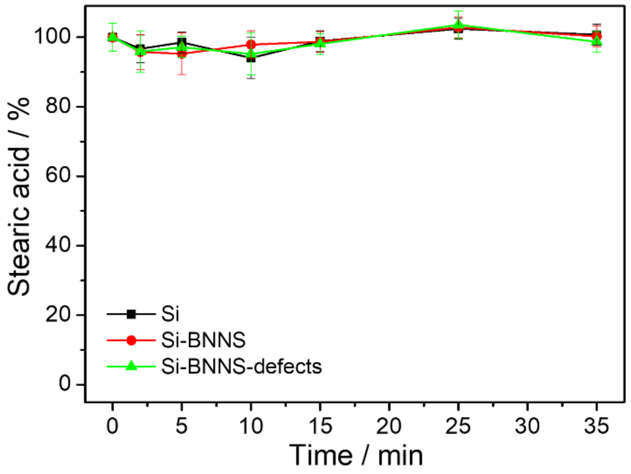

Figure S3. Photodegrading results of stearic acid deposited on bare silicon substrate with BNNS.

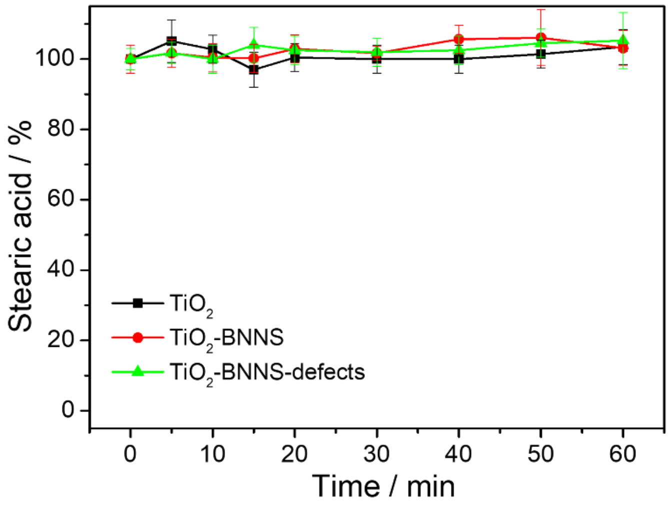

Figure S4. Photodegrading results of stearic acid under the catalysis of the films after thermally treating at 350 °C driven by blue light of 450 nm.

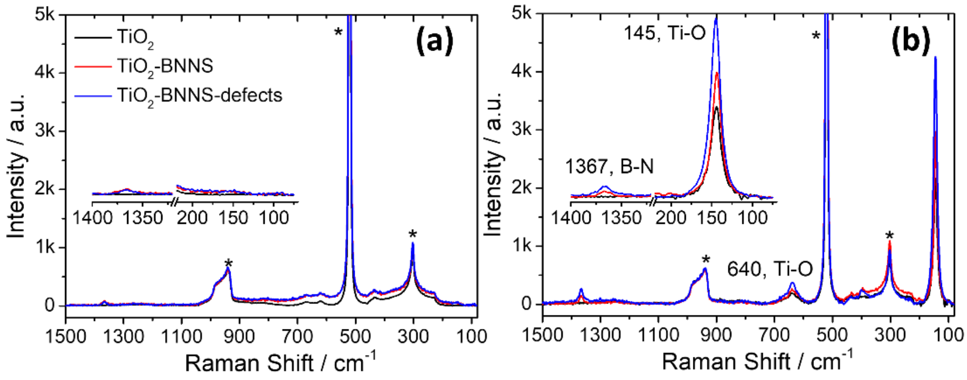

Figure S5. Raman spectra of TiO_2, TiO_2-BNNS, and TiO_2-BNNS(d) mesoporous films after thermal treatment at (a) 300 and (b) 400 °C. Insets show the enlarged spectra in the 1400 – 1320 cm^{-1} and 220 – 70 cm^{-1} ranges. Raman signals at 300, 520, and 940 cm^{-1} are due to the silicon wafer substrate (see symbol *).

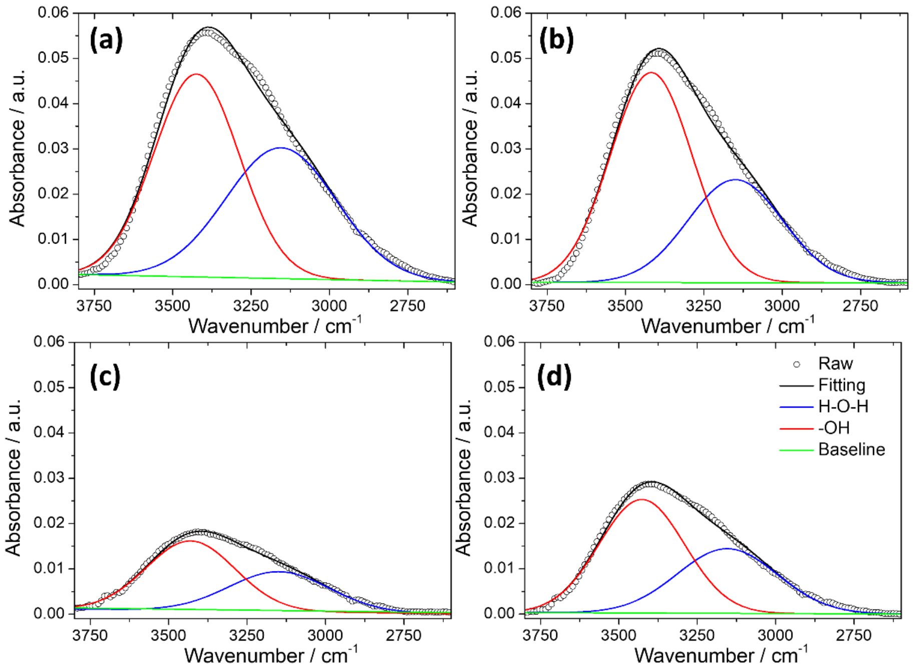

Figure S6. FTIR absorption spectra and their the deconvoluted components in the 3800 – 2600 cm^{-1} range of the TiO_2-BNNS-defects films treated at (a) 300 °C, (b) 350 °C, and (c) 400 °C, as well as (d) TiO_2 film treated at 400 °C.

Table S1. The relative intensities of the deconvoluted infrared bands H-O-H and Ti-OH according to **Figure S6**.

Films	at 300 °C	at 350 °C	at 400 °C	
			With BN	Without BN
H-O-H	45.5%	30.7%	12.5%	20.4%
Ti-OH	54.5%	54.3%	19.%	30.6%

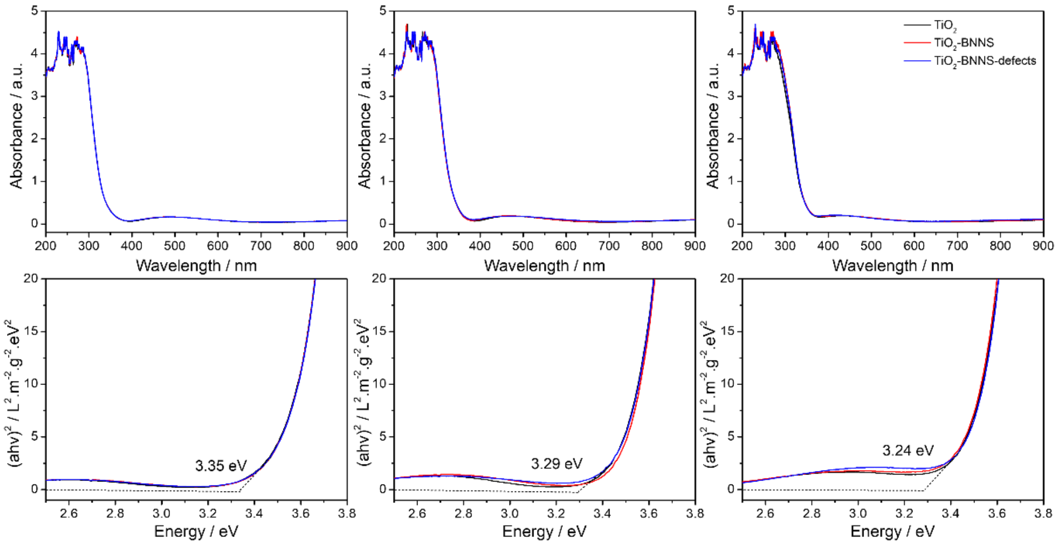

Figure S7. UV-Vis absorption spectra (on top) and E_g evaluation using TAUC equation (on bottom) of the nanocomposite films. The data of left to right are from the films firing at 300, 350, and 400 °C, respectively.

Table S2. Refractive index (n) at 633 nm of TiO_2 nanocomposite films tested by spectroscopic ellipsometry.

Films	300 °C	350 °C	400 °C
TiO_2	1.946	1.970	1.987
TiO_2-BNNS	1.960	1.955	2.018
TiO_2-BNNS-defect	1.960	2.002	2.026

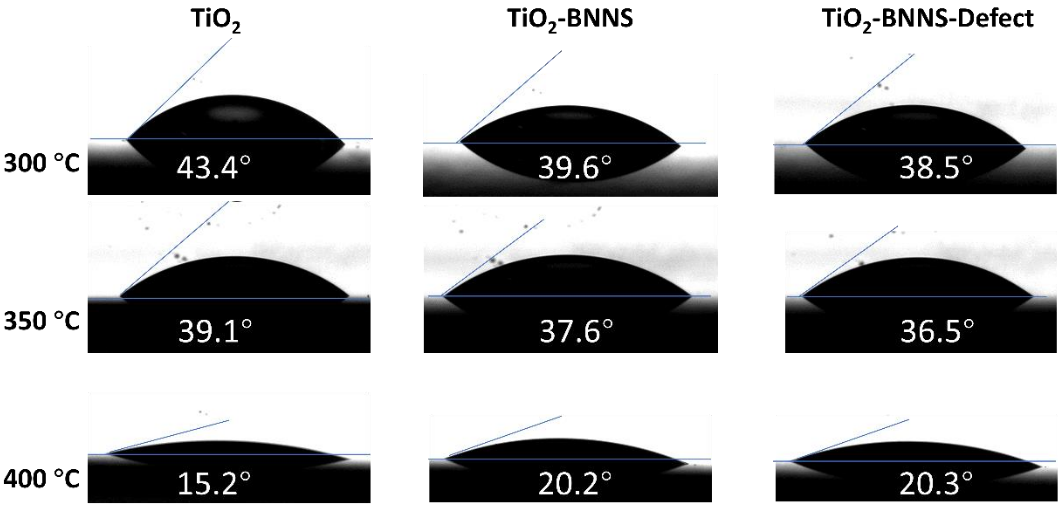

Figure S8. Contact angle images using water droplets deposited on the nanocomposite films.
